# Female Mucopolysaccharidosis IIIA Mice Exhibit Hyperactivity and a Reduced Sense of Danger in the Open Field Test

**DOI:** 10.1371/journal.pone.0025717

**Published:** 2011-10-18

**Authors:** Alex Langford-Smith, Kia J. Langford-Smith, Simon A. Jones, Robert F. Wynn, J. E. Wraith, Fiona L. Wilkinson, Brian W. Bigger

**Affiliations:** 1 Stem Cell and Neurotherapies, School of Biomedicine, Faculty of Medical and Human Sciences, University of Manchester, Manchester, United Kingdom; 2 Genetic Medicine, St. Mary's Hospital, Manchester, United Kingdom; 3 Blood and Marrow Transplant Unit, Royal Manchester Children's Hospital, Manchester, United Kingdom; Nathan Kline Institute and New York University School of Medicine, United States of America

## Abstract

Reliable behavioural tests in animal models of neurodegenerative diseases allow us to study the natural history of disease and evaluate the efficacy of novel therapies. Mucopolysaccharidosis IIIA (MPS IIIA or Sanfilippo A), is a severe, neurodegenerative lysosomal storage disorder caused by a deficiency in the heparan sulphate catabolising enzyme, sulfamidase. Undegraded heparan sulphate accumulates, resulting in lysosomal enlargement and cellular dysfunction. Patients suffer a progressive loss of motor and cognitive function with severe behavioural manifestations and premature death. There is currently no treatment. A spontaneously occurring mouse model of the disease has been described, that has approximately 3% of normal enzyme activity levels. Behavioural phenotyping of the MPS IIIA mouse has been previously reported, but the results are conflicting and variable, even after full backcrossing to the C57BL/6 background. Therefore we have independently backcrossed the MPS IIIA model onto the C57BL/6J background and evaluated the behaviour of male and female MPS IIIA mice at 4, 6 and 8 months of age using the open field test, elevated plus maze, inverted screen and horizontal bar crossing at the same circadian time point. Using a 60 minute open field, we have demonstrated that female MPS IIIA mice are hyperactive, have a longer path length, display rapid exploratory behaviour and spend less time immobile than WT mice. Female MPS IIIA mice also display a reduced sense of danger and spend more time in the centre of the open field. There were no significant differences found between male WT and MPS IIIA mice and no differences in neuromuscular strength were seen with either sex. The altered natural history of behaviour that we observe in the MPS IIIA mouse will allow more accurate evaluation of novel therapeutics for MPS IIIA and potentially other neurodegenerative disorders.

## Introduction

Mucopolysaccharidosis IIIA (MPS IIIA, OMIM #252900), or Sanfilippo Type A, is an autosomal recessive lysosomal storage disorder that affects 0.82 in 100,000 live births in the United Kingdom [1]. The disease is characterised by severe and progressive loss of cognitive and motor functions, behavioural difficulties and eventually death in the second decade of life, although the severity and progression of the disease varies widely [1], [2], [3]. MPS IIIA is caused by mutations in the *SGSH* gene that result in deficiency of the N-sulfoglucosamine sulfohydrolase enzyme (sulfamidase, EC 3.10.1.1) and subsequent accumulation of undegraded heparan sulphate, lysosomal enlargement and cellular and organ dysfunction [4], [5], [6], [7]. Patients exhibit progressive neurodegeneration and behavioural problems including hyperactivity, a reduced sense of danger, aggression and sleep disturbances [7], [8], [9], [10], [11], [12].

Although there are no current therapies, several strategies are in development for MPS IIIA or the phenotypically indistinguishable MPS IIIB disease, including substrate reduction therapy [13], [14], [15], intrathecal enzyme replacement therapy [16], [17], [18], [19], [20] and gene therapy with lentiviral [21], adenoviral [22] or adeno-associated-viral [23] vectors. Most strategies make use of the ability of exogenous enzyme to complement affected cells, however the presence of the blood brain barrier limits efficient enzyme distribution. Biomarkers for MPS IIIA or related diseases are still in development [24], [25], thus the accurate evaluation of neurodegeneration using behavioural phenotyping in the mouse model of disease is paramount. A spontaneously occurring mouse model of MPS IIIA on a mixed 129SvJ, C57BL/6, SJL, and CD1 background has been previously described with around 3% of normal enzyme activity and exhibiting many of the features observed in patients [26]. The MPS IIIA mice exhibit severe neuropathology characterised by enlarged lysosomes, primary storage of HS, secondary storage of GM2 and GM3 gangliosides and cholesterol and chronic neuroinflammation [19], [23], [26], [27], [28]. The MPS IIIA mice are euthanized between 9 and 12 months of age due to urine retention, a phenotype not seen in the patients but also seen in MPS IIIB mice [29]. The MPS IIIA mouse model has been backcrossed to the C57BL/6 background by Professor John Hopwood's Lysosomal Diseases Research Unit [30] and to the C57BL/6J background by Jackson laboratories [31].

Although the behaviour of the MPS IIIA mouse model has been evaluated in the open field test at several ages, using different sexes, by several groups and on different strain backgrounds, there is a remarkable amount of variability in the outcome of the test as outlined in Table 1.

**Table 1:** Summary of the significant differences in the open field activity of MPS IIIA mice in the literature.

**Paper**		Hemsley *et al.*		Hemsley *et al.*	Lau *et al.*		Lau *et al.*	McIntyre *et al.*	Crawley *et al.*		* Hemsley et al.	* Hemsley et al.	* McIntyre et al.	* Fraldi et al.	This Study	
**Year**		2005		2007	2008		2010	2010	2006		*2007*	*2009*	*2010*	*2007*		
**Background**		Mixed		Mixed	C57BL/6		C57BL/6	C57BL/6	C57BL/6		*Mixed*	*C57BL/6*	*C57BL/6*	*C57BL/6*	C57BL/6J	
**Measure**		Line Crossing		Line Crossing	Zone Entries		Path Length	Line crossing	Line Crossing		*Line Crossing*	*Path Length*	*Line crossing*	*Path Length*	Path Length	
**Sex**		Male	Female	Male	Male	Female	Male	Male	Male	Female	*Male*	*Male*	*Male*	*Male*	Male	Female
**Age (Weeks)**	**3**	Hyper	Hyper		NS	NS										
	**4**								NS	Hypo						
	**5**				NS	NS										
	**6**	Hypo	NS	Hypo					NS	NS						
	**8**								NS	NS	NS* +Hypo*					
	**10**	NS	NS		Hypo	NS			NS	NS				NS*		
	**12**								Hyper	NS	NS* +Hypo*	Hypo*				
	**15**	Hypo	NS		NS	NS	Hypo		NS	NS				NS*		
	**16**														NS	Hyper
	**18**				Hypo	NS			NS	NS	Hypo*			NS*		
	**20**	NS	NS					Hypo					NS*			
	**21**													Hyper*		
	**22**				Hypo	NS			NS	Hyper						
	**24**							NS					NS*		NS	Hyper
	**25**								NS	NS						
	**28**							NS					NS*			
	**32**								NS	Hyper					NS	Some Hyper
	**40**	NS	NS						NS	NS						

Hyper indicates significant hyperactive behaviour and hypo indicates significant hypoactive behaviour in the open field test. NS indicates no significant difference and * indicates mice that have recieved intracranial injections. Control injections contained: 2.5 µl of 50 mM sodium acetate, 100 mM sodium chloride at pH 5.0 in Hemsley *et al* 2007, 4 µl 10 mM sodium phosphate, 140 mM sodium chloride pH 7.0 in Hemsley *et al.* 2009, 5 µl 0.9% (w/v) sodium chloride in McIntyre *et al.* 2010 and 1 µl GFP adeno-associated viral vector in Fraldi *et al.* 2007. The work of Hemsley et *al.* 2007 contained multiple control treated groups and where there were differences between groups, both have been shown. Where female mice are not mentioned or cells are blank no testing was performed.

Using the mixed background mouse, at many of the time points measured, no differences are observed between either male or female WT and MPS IIIA mice, with initial findings of hyperactivity and later hypoactivity in males [18], [32]. After back crossing the MPS IIIA mice on to a C57BL/6 background, the behaviour was extensively studied by Crawley *et al.* at different time points. Hyperactivity in males was observed at 12 weeks of age, and females at 22 and 32 weeks of age, although these mice were not naively tested [30]. At most time points, no differences were seen. However male, but not female, MPS IIIA mice were shown to be hypoactive at some time points in the work of Lau *et al* [33] and male MPS IIIA mice were hypoactive at 15 weeks of age in a more recent paper by Lau *et al.* [34]. Hypoactivity in male mice was also observed at some time points in McIntyre *et al.* [21]. In columns 7, 8, 9 and 10 of Table 1 (marked as bold) we have also presented markedly variable outcomes of observations of locomotor behaviour of MPS IIIA and WT mice that received control intracranial injections [18], [21], [23], [35].

Patients with MPS IIIA are believed to have a reduced sense of danger, which can be inferred in mice by the amount of time spent in the centre of the open field test or by use of the elevated plus maze test, both of which can be used to measure their tendency to avoid open spaces and remain close to cover (thigmotaxis). No difference in the time in centre of the open field was detected by Lau *et al.* [33], however this could be due to the small size of the open field used. In the elevated plus maze, male MPS IIIA mice were reported to display reduced anxiety with a greater proportion of their path length (distance travelled) spent in the open arms at some time points but not others [33], [34].

Overall, there are discrepancies in behaviour of this mouse model that could be due to gender differences, the age of testing and the methodology used to perform the tests. Therefore we have attempted to perform a standardised analysis of MPS IIIA mice by first backcrossing them to the C57BL/6J background for over 10 generations and subsequently testing a cohort of MPS IIIA and WT mice at the same circadian time point at 4, 6 and 8 months (16, 24 and 32 weeks) of age in the 60 minute open field test, the elevated plus maze, as well as several other neuromuscular evaluations that we have previously shown to be effective for phenotyping MPS IIIB mice [36]. The female MPS IIIA mice were hyperactivity, had a recued sense of danger and no neuromuscular differences. The open field test performed at the same point in the circadian rhythm was a consistent, sensitive and reliable behavioural test for the evaluation of novel therapeutic strategies in MPS IIIA mice.

## Methods

### Mouse maintenance

The MPS IIIA mouse colony was maintained through heterozygous breeding at the University of Manchester, all procedures were ethically approved by the University of Manchester Ethical Review Process Committee and in accordance with the UK Home Office regulations under project licence PPL 40/3117. Mice were housed in individually ventilated cages, had access *ad libitum* to food and water and were in a 12 hour light and dark cycle. Male mice were singly housed at 14 weeks of age due to aggressive tendencies but female mice remained housed in groups of 4–6. The MPS IIIA mice (B6.Cg-Sgsh^mps3a^) have been backcrossed from the original mixed 129SvJ, C57BL/6, SJL, and CD1 background [26] onto the C57BL/6J background by more than 10 generations of backcrossing with C57BL/6J mice (Harlan, UK) and were maintained by heterozygous breeding. WT and MPS IIIA littermates have been used in all behavioural experiments.

### Genotyping MPS IIIA mice

MPS IIIA mice have a G to A mutation in the *SGSH* gene which removes a *Msp*1I restriction enzyme digestion site. Genotyping is performed by PCR amplification of DNA followed by *Msp*1I digestion and observation of the size of DNA fragments produced. Genomic DNA was extracted from ear punches using the GenElute Mammalian Genomic DNA Miniprep Kit (Sigma Aldrich) following the manufacturer's instructions and *SGSH* was amplified by PCR using the forward primer 5′ GTGTTCCCTGCCTGCTCAC 3′ and reverse primer 5′ CCAGTCCCCTCATCCCACTA 3′. The DNA was digested with *Msp*1I (New England Biolabs, UK) and the DNA fragments were separated by 2% agarose gel electrophoresis. The genotype was determined from the pattern of DNA fragments; wild type (WT) reveals 199 bp, 118 bp and 78 bp fragments, mutant (MUT) 317 bp and 78 bp fragments and heterozygous (HET) 317 bp, 199 bp, 118 bp and 78 bp.

### Behavioural testing

At 4, 6 and 8 months (16, 24 and 32 weeks) of age, the same cohorts of 10 male WT and 10 male MPS IIIA mice and 10 female WT and 11 MPS IIIA mice were analysed with the following behavioural tests. 1.5 hours into the 12 hour light phase the mice were dropped into the centre of an open field arena (width 450 mm, depth 450 mm, height 500 mm) made of matt white acrylic. The behaviour was recorded for 60 minutes using a digital camcorder (Sony) and analysed using Top Scan software (Clever Sys. Inc., USA). The path length, frequency (number of times) and duration of rapid exploratory behaviour (speed >100 mm/s), frequency and duration of immobility (speed <5 mm/s) and frequency of entering the centre and duration in the centre (75 mm from each edge) was analysed. Rearing in the open field was analysed by counting the number of unsupported rears (front paws off the floor) and supported rears (front paws on the wall) in the first 10 minutes. The same researcher performed all experiments and was blinded to genotype.

After a 30 minute rest, the mice were placed onto the end of an open arm of an elevated plus maze. The maze was constructed of matt white acrylic, comprised of four 500 mm long by 100 mm wide arms, two of which were enclosed by 500 mm high walls, and was raised on a stand 500 mm off the floor. Mice were placed so they faced towards the centre of the maze and were given 10 minutes to explore the maze before returning to a cage. The maze was cleaned using 70% ethanol. 30 minutes later the mice were tested for a second time, in the same manner. The results were analysed using Top Scan software (Clever Sys) to examine the amount of time spent on the open arm, the percentage of path length in the open arm, and the percentage of open arm entries.

30 minutes later the inverted screen test was performed, as described previously [36]. In brief, at 4, 6 and 8 months of age the mouse was placed on a 470 mm square with a 13 mm square wire mesh. The screen was then rotated through 180° over 1–2 seconds. The mouse was then suspended upside down over a padded surface; the rear leg moves were counted and the duration suspended was recorded up to 2 minutes.

One hour later the horizontal bar test was performed as described previously [14], [36]. In brief, a 2 mm diameter, 300 mm long metal wire was secured between 2 posts, 320 mm above a padded surface. The mouse was allowed to grip the centre of the wire and the time taken to fall or to reach the side was recorded up to 2 minutes. The test was repeated three times as a training run followed by a 10 minute rest before three test runs. The results were scored as follows: crossing the bar in x seconds was scored as 240-x, remaining on the bar was scored as 120 and falling off the bar after y seconds was recorded as y.

### Urine retention

Upon sacrifice 9 male WT, 8 male MPS IIIA, 7 female WT and 8 female MPS IIIA mice at 8 months (32 weeks) of age were dissected and the urine was removed from the bladder and volume measured using an insulin syringe (BD).

### Statistical analysis

Statistical analysis was performed using JMP software (SAS Institute Inc, Cary, NC, USA) and analysed by MANOVA with repeated measures for overall significances and by one or two way ANOVA, as appropriate, with Tukey post hoc tests at individual time points. Significance was set at p≤0.05. For the MANOVA analysis the Genotype significance determines if there is a difference between WT and MPS IIIA mice irrespective of the age of the mice, the Time significance determines if there is a change in the behaviour of the mice at different ages irrespective of the genotype and the Genotype*Time significance determines if MPS IIIA mouse behaviour changes over time in a different manner to WT mice.

## Results

**Figure 1:**
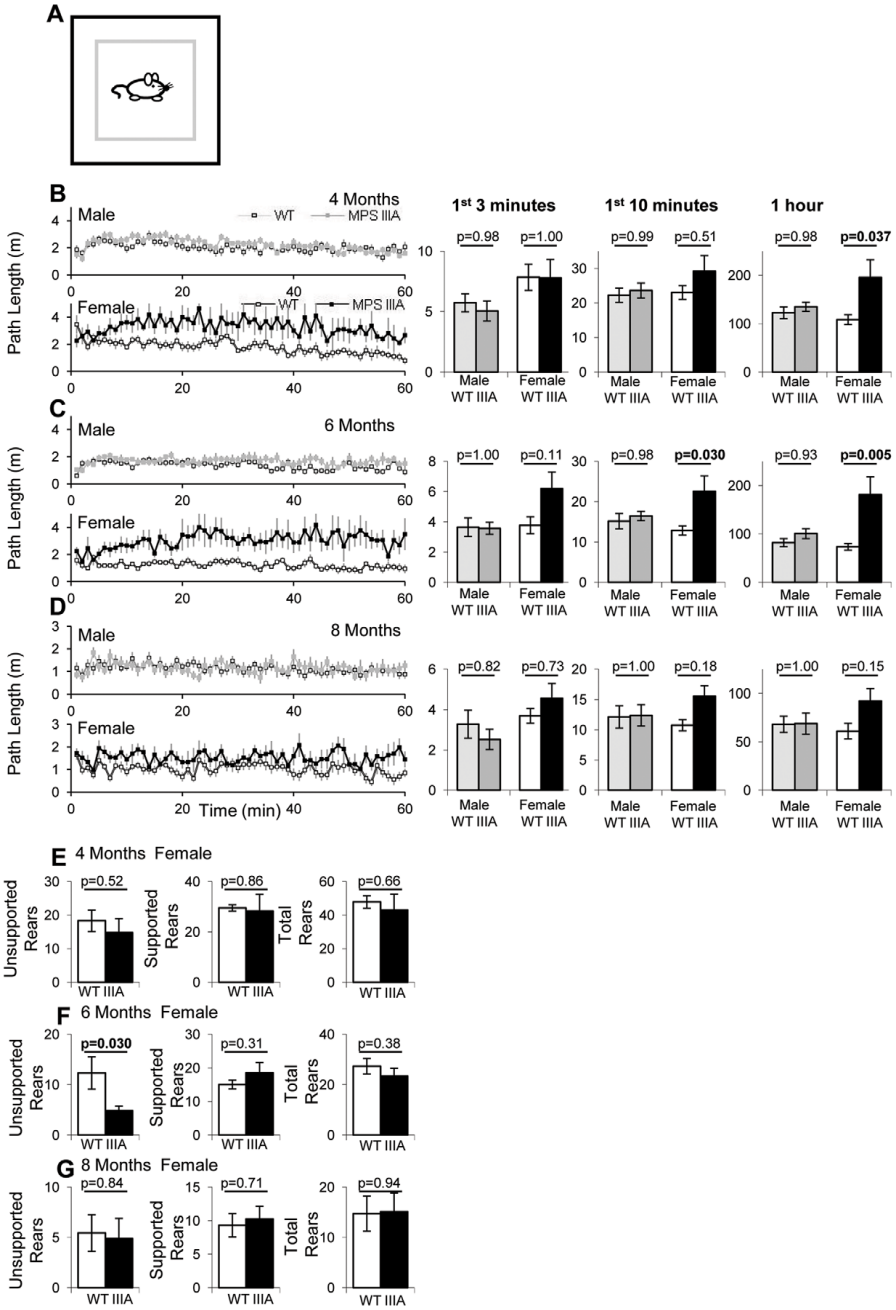
Open field path length and rearing. At 4, 6 and 8 months (16, 24 and 32 weeks) of age, 10 WT male (light grey squares), 10 MPS IIIA male (dark grey squares), 10 WT female (white squares) and 11 MPS IIIA female (black squares) were placed in the open field and the behaviour was recorded for 60 minutes (A). The results of the open field behaviour are presented as a 60 minute period with the average of every minute presented and as a bar chart of the first 3 minutes, first 10 minutes and the whole hour. Error bars represent the standard error of the mean (SEM). p values were calculated by 2 Way ANOVA. The mean average path length in Metres at 4 (B), 6 (C) and 8 (D) months have been presented. The number of unsupported, supported and total rears in the first 10 minutes at 4 (E), 6 (F) and 8 (G) months of age have also been presented.

Cohorts of male and female, WT and MPS IIIA mice (n = 10–11 per group) were monitored in a 60 minute open field test as outlined in Figure 1A. The path length minute by minute over 60 minutes is shown at 4 months (16 weeks) in Figure 1B, 6 months (24 weeks) in Figure 1C and at 8 months (32 weeks) in Figure 1D. The behaviour in the first 3 minutes, 10 minutes and 60 minutes is presented to allow comparison against published data and to identify the minimum amount of time required for statistically significant analyses. At 4 months of age, female MPS IIIA mice were significantly hyperactive and travel almost twice as far as the WT mice over 60 minutes (p = 0.037; Figure 1B). Examining the behaviour over the first 3 minutes showed no difference and no trend in female behaviour, but over 10 minutes there was a non-significant trend towards hyperactivity. No difference was found in the path length of male WT or MPS IIIA mice at 4 months.

At 6 months of age the same female MPS IIIA mice demonstrated increased path length with significant differences after 10 minutes (p = 0.030) and greater differences after 60 minutes (p = 0.005; Figure 1C). No difference was detected between male WT and MPS IIIA mice (Figure 1C).

At 8 months of age there was a trend for the female mice to be hyperactive but no significant differences were found after 3 (p = 0.73), 10 (p = 0.15) or 60 minutes (p = 0.18; Figure 1D). Similarly, no significant differences were found with male mice after 3, 10 or 60 minutes. Overall, using MANOVA repeated measure analysis, a significant genotype difference (p = 0.011), time difference (p = 1.2×10^−7^) and a time*genotype difference (p = 0.021) were found. This indicates that overall, MPS IIIA mice are significantly more hyperactive than WT mice, that this changes with time and that MPS IIIA and WT mice change their behaviour in a different manner over time.

The number of unsupported rears (front paws off the floor), supported rears (front paws on a wall) and total rears were counted manually in the first 10 minutes of the open field test. The only significant change observed was a decrease in the number of unsupported rears by female MPS IIIA mice at 6 months of age (Figure 1F). However, given that there were no other significant genotype effects, this may be just a chance occurrence (Figure 1E and G). MANOVA repeated measure analysis of the data showed no significant genotype effect in the three rearing measures, but there was a significant decline in all measures with time (unsupported; p = 0.004, supported and total p = 0.00003) irrespective of genotype.

**Figure 2:**
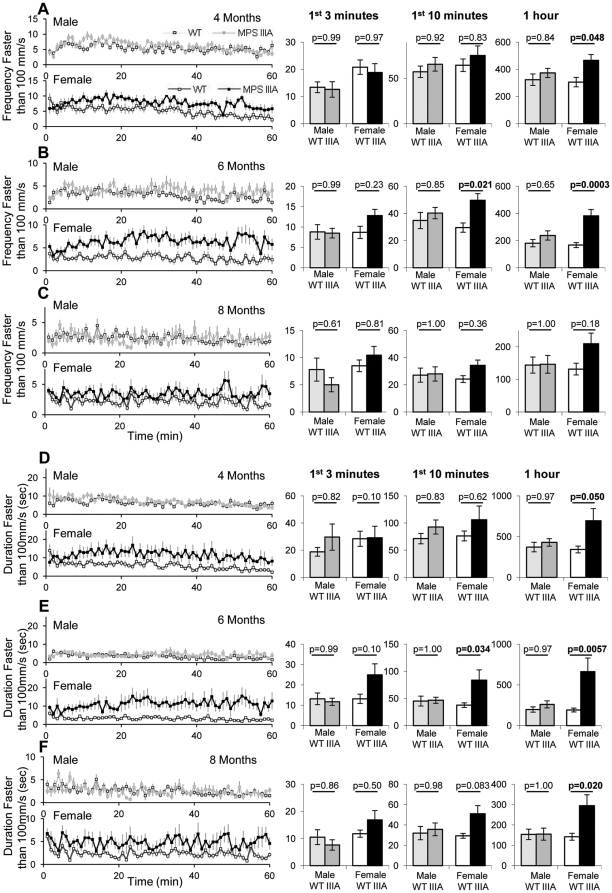
Open field rapid exploratory behaviour. At 4, 6 and 8 months of age 10 WT male (light grey squares), 10 MPS IIIA male (dark grey squares), 10 WT female (white squares) and 11 MPS IIIA female (black squares) were placed in the open field and the behaviour was recorded for 60 minutes. The results of this test are presented as a 60 minute period with the average of every minute presented and as a bar chart of the first 3 minutes, first 10 minutes and the whole hour. Error bars represent the SEM. p values were calculated by 2 Way ANOVA. The frequency of moving faster than 100 mm/s at 4 (A), 6 (B) and 8 (C) months and the duration spent moving faster than 100 mm/s at 4 (D), 6 (E) and 8 (F) months of age have been presented.

Rapid exploratory motion was analysed by measuring the frequency and duration of speed over 100 mm/s (Figure 2A–F). At 4 months of age, female MPS IIIA mice show significant increases in both the frequency (p = 0.048; Figure 2A) and duration (p = 0.05; Figure 2D) of rapid exploration over 60 minutes. However, no significant differences were observed after 3 or 10 minutes, although a trend was detected after 10 minutes (Figure 2A). No significant differences were found between male MPS IIIA or WT mice at 4 months of age.

At 6 months of age, there was a trend towards increased frequency (Figure 2B) and duration (Figure 2E) of rapid exploratory motion after 3 minutes in the female MPS IIIA mice (p = 0.23, p = 0.10). After 10 (p = 0.021, p = 0.034) and 60 minutes (p = 0.0003, p = 0.0057), female MPS IIIA mice had significantly increased frequency (Figure 2B) and duration (Figure 2E) of rapid exploratory motion over 100 mm/s. No significant differences were observed between male WT and MPS IIIA mice. This rapid exploratory behaviour is visible in Video S1 which shows the median female WT and MPS IIIA mouse at 6 months of age at 4 times normal speed.

At 8 months of age, female MPS IIIA mice show a trend towards increased frequency of rapid exploratory motion over 60 minutes (p = 0.18) (Figure 2C), with a trend to increase in duration after 10 minutes (p = 0.083) and a significant increase after 60 minutes (p = 0.02) (Figure 2F). Male MPS IIIA and WT mice were indistinguishable. Using MANOVA repeated measure analysis, there was a significant difference between WT and MPS IIIA mice over all three time points for frequency (Genotype; p = 0.004) and duration (Genotype; p = 0.013) of rapid exploratory motion. There was a significant decline in frequency (Time; p = 9.7×10^−15^) and duration (Time, p = 4.5×10^−8^) of rapid exploratory motion with time and also between genotypes over time for frequency (Time*Genotype p = 0.00005) and duration (Time*Genotype, p = 0.036).

**Figure 3:**
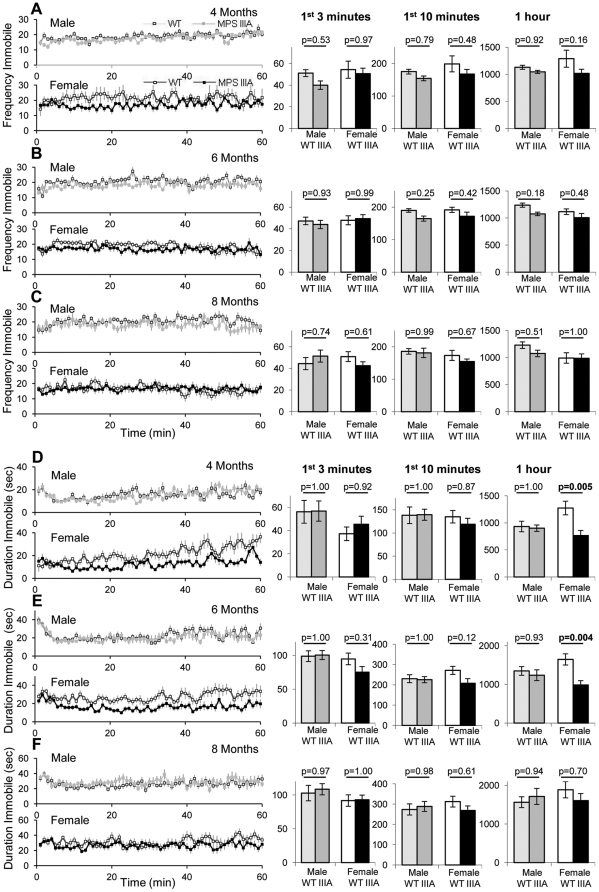
Open field immobile behaviour. At 4, 6 and 8 months of age 10 WT male (light grey squares), 10 MPS IIIA male (dark grey squares), 10 WT female (white squares) and 11 MPS IIIA female (black squares) were placed in the open field and the behaviour was recorded for 60 minutes. The results of this test are presented as a 60 minute period with the average of every minute presented and as a bar chart of the first 3 minutes, first 10 minutes and the whole hour. Error bars represent the SEM. p values were calculated by 2 Way ANOVA. The frequency of not moving at 4 (A), 6 (B) and 8 (C) months and the duration spent not moving at 4 (D), 6 (E) and 8 (F) months of age have been presented.

The frequency and duration the mice spent immobile was also recorded (Figure 3A–F). There were no significant differences in frequency of immobility between either male or female MPS IIIA and WT mice at any time point. However, female WT mice had significantly increased duration of immobility over 60 minutes at 4 (p = 0.005) and 6 months (p = 0.004) but not at 8 months (p = 0.70). Male MPS IIIA or WT mice had indistinguishable duration of immobility at all time points measured. Using MANOVA repeated measures there was an overall increase in the frequency of WT immobility over MPS IIIA mice over the three time points (Genotype; p = 0.027), but no change with time (Time; p = 0.543), or genotype with time interaction (Genotype*Time; p = 0.636). MANOVA repeated measures analysis of duration of immobility demonstrated a significant genotype effect over the three time points (Genotype; p = 0.05), that changes significantly with time (Time; p = 1.5×10^−9^) and changes differently with time between the two genotypes (Genotype*Time; p = 0.030).

**Figure 4:**
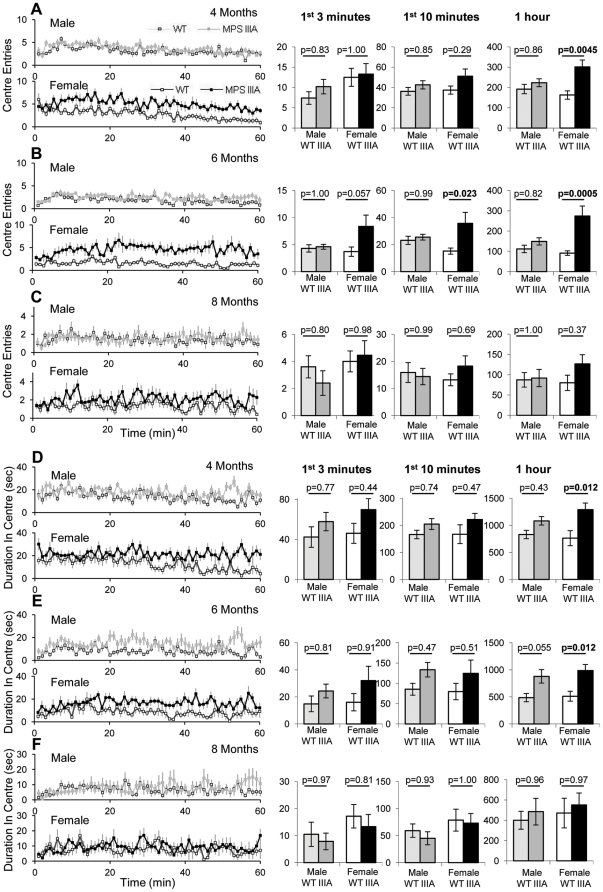
Open field sense of danger behaviour. At 4, 6 and 8 months of age 10 WT male (light grey squares), 10 MPS IIIA male (dark grey squares), 10 WT female (white squares) and 11 MPS IIIA female (black squares) were placed in the open field and the behaviour was recorded for 60 minutes. The results of this test are presented as a 60 minute period with the average of every minute presented and as a bar chart of the first 3 minutes, first 10 minutes and the whole hour. Error bars represent the SEM. p values were calculated by 2 Way ANOVA. The frequency of entering the centre at 4 (A), 6 (B) and 8 (C) months and the duration spent in the centre at 4 (D), 6 (E) and 8 (F) months of age have been presented.

Mice being a prey species tend to display thigmotaxis, remaining close to the sides of an open field arena. Increased frequency and duration of time spent in the centre of the open field test therefore demonstrates reduced thigmotaxis and this is commonly considered to be a measure of reduced anxiety or reduced sense of danger that the animal experiences [37]. Male MPS IIIA and WT mice showed no significant differences in frequency of centre entries at any age (Figure 4A, B and C). At 4 months of age, female MPS IIIA mice showed significantly increased centre entries at 60 minutes (p = 0.0045). At 6 months female MPS IIIA mice entered the centre significantly more at 3, 10 and 60 minutes (p = 0.057, p = 0.023, p = 0.0005; Figure 4B). At 8 months of age female MPS IIIA showed no significant increases in centre entries (p = 0.37)(Figure 4C). Using MANOVA MPS IIIA mice showed significantly increased centre entries (Genotype; p = 0.003), which changed significantly over time (Time; p = 2.3×10^−11^) and between genotypes over time (Genotype*Time; p = 0.0013).

The duration spent in the centre has also been analysed. At 4 months of age, female MPS IIIA mice spent significantly more time in the centre than WT, after 60 minutes (p = 0.012; Figure 4D). Males were not significantly different. At 6 months of age female MPS IIIA mice had increase duration in the centre after 60 minutes (p = 0.012) and showed an almost significant trend in male mice (p = 0.055; Figure 4E). At 8 months of age there are no significant differences between genotypes (Figure 4F). By MANOVA repeated measure analysis MPS IIIA mice spend significantly increased duration in the centre area (Genotype; p = 0.001), this changes significantly over time (Time; p = 2.5×10^−6^) and between genotypes over time (Time*Genotype; p = 0.005).

**Figure 5:**
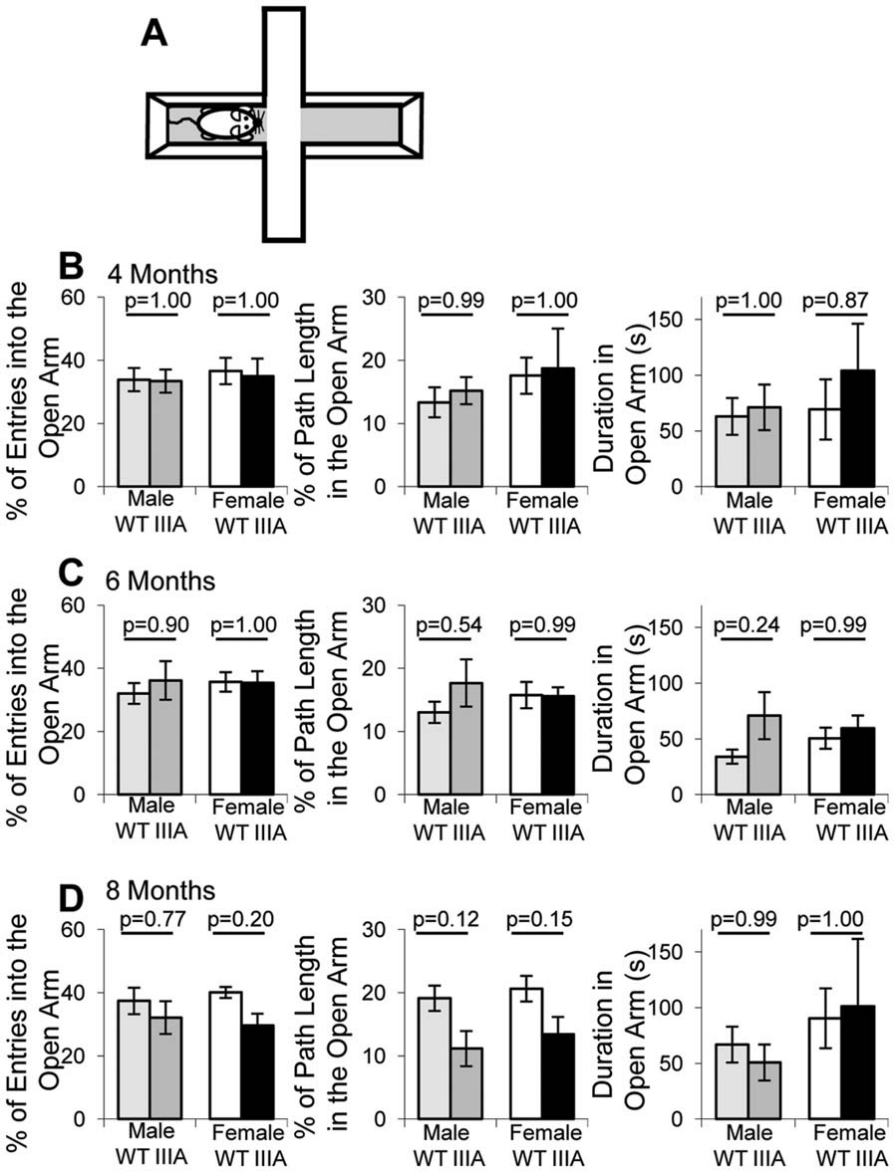
Elevated plus maze behaviour. At 4, 6 and 8 months of age 7–11 WT male (light grey bars), MPS IIIA male (dark grey bars), WT female (white bars) and MPS IIIA female (black bars) were placed on the elevated plus maze (A) and the behaviour was recorded for 10 minutes. After a 30 minute rest, the test was repeated. The results of the first test are presented as the mean of each measure with error bars representing the SEM. p values were calculated by 2 Way ANOVA. The following measures have been presented; the percentage of total entries that were into the open arm, the percentage of the path length in the open arm and the duration spent on the open arm at 4 (B), 6 (C) and 8 (D) months of age.

Thirty minutes after behavioural analysis in the open field, the same cohort of mice were tested on an elevated plus maze (Figure 5A) for ten minutes, followed by a 30 minute rest and another 10 minute trial. Several parameters were measured (frequency of entering open arm, percentage of entries into the open arm, path length in open arm, percentage of path length in open arm and time in open arm) but none reached significance. Here we have presented the percentage of entries to the open arms, percentage of path length in the open arms and duration in the open arms as measure of anxiety (Figure 5B, C, D), which is representative of many of the other measures. At 4, 6 and 8 months of age there was no significant difference in male or female WT or MPS IIIA open arm entries (Figure 5B, C, D). At 4, 6 and 8 months there was no difference between WT and MPS IIIA mice in the repeat elevated plus maze and no difference between first and second elevated plus maze tests except that in the second test, all the mice performed fewer entries and had a shorter path length (Figure S1A–C). Statistical analysis by MANOVA showed no significant difference in genotype, or time but there was a significant difference in how the genotypes behaved over time in the percentage of path length in the open arm (p = 0.019) as the MPS IIIA mouse path length decreased at 8 months but the WT increased.

**Figure 6:**
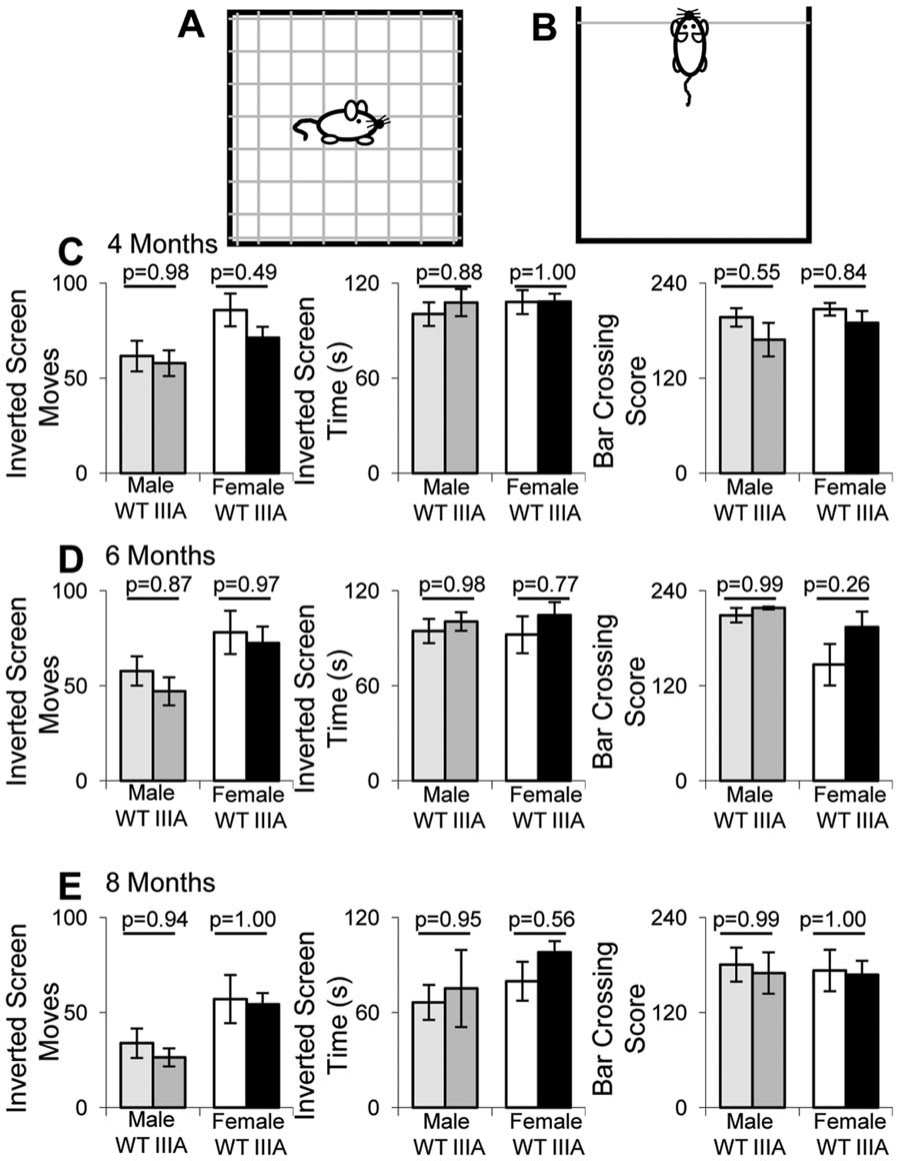
Neuromuscular behaviour. At 4, 6 and 8 months of age 8–11 WT male (light grey bars), MPS IIIA male (dark grey bars), WT female (white bars) and MPS IIIA female (black bars) were placed on the inverted screen test (A) and horizontal bar crossing test (B). The number of rear leg moves, the time spent on the inverted screen and the bar crossing score are presented at 4 (C), 6 (D) and 8 (E) moths of age. Error bars represent the SEM and p values were calculated by 2 Way ANOVA.

The inverted screen test (Figure 6A) and horizontal bar crossing test (Figure 6B) were also performed. The inverted screen test measures neuromuscular strength and the bar crossing test measures both neuromuscular strength and motor coordination. The bar crossing test showed no significant differences between WT and MPS IIIA of either sex at 4, 6 or 8 months of age (Figure 6C–E). Over time, there was a significant decrease in the number of moves and time spent on the inverted screen (p = 1.7×10^−6^, p = 1.2×10^−5^), and a significant sex difference in the number of moves with females moving more (p = 0.004). Bar crossing showed no genotype effect and no time effect but there was a significant Time*Sex effect (p = 0.044), as the female score decreased over time but the male score increased at 6 months and then decreased at 8 months.

## Discussion

We have backcrossed the mixed background MPS IIIA mouse model onto the C57BL/6J background and characterised a behavioural phenotype that can be used to accurately distinguish female MPS IIIA mice from WT littermates. Our findings clearly show that male mice show no significant difference to WT littermates in the open field test or elevated plus maze, whilst female MPS IIIA mice demonstrate characteristic hyperactive behaviour initially at 4 months (16 weeks) and more strongly at 6 months (24 weeks), which declines again at 8 months (32 weeks) of age. We also show that these mice have a reduced thigmotaxis at 4 and 6 months of age which would indicate a reduced sense of danger. Hyperactivity and a reduced sense of danger are observed in the behavioural phenotype of the MPS IIIA patients [1], [7], [10], [11].

In this study we observed no significant behavioural differences between WT and MPS IIIA male mice. This is in contrast to Hemsley *et al.* 2005 where hyperactivity was initially observed at 3 weeks, hypoactivity at 6 and 15 weeks and no changes at 10, 20 and 40 weeks in male mixed background mice [32]. Lau *et al.* 2008 used male MPS IIIA mice that had been back crossed onto the C57BL/6 background and observed no differences at 3, 5 and 15 weeks, with significant hypoactivity at 10, 18 and 22 weeks [33] but subsequently observed hypoactivity in 15 week old male mice [22]. Crawley *et al.*, using male MPS IIIA mice on the same C57BL/6 background, found no changes at 4, 6, 8, 10, 15, 18, 22, 25, 32 and 40 weeks and only observed hyperactivity at 12 weeks [30]. Finally, using male mice independently crossed onto the C57BL/6J background by Jackson laboratories, hypoactivity was observed in MPS IIIA mice at 20 weeks of age by McIntyre *et al.* but no difference was found at 24 or 32 weeks [38]. Clearly, it is hard to obtain consistent behavioural readouts from male mice in this context and it is always difficult to know if hypoactivity could be confounded by any parameter affecting the mouse's physical ability to move. We find male MPS IIIA mice to be very aggressive and are unable to keep them group housed, which is likely to change their behaviour. Singly housing male C57BL/6 mice has been shown to increase locomotor activity and reduce anxiety [39] or alternatively in other researchers hands to have no effect [40]. At the very least, it certainly leads to weight gains, which will restrict movement, and this may confound reliable open field measures. Additionally we find that all MPS IIIA mice retain urine over time, with male mice retaining significantly more urine at 8 months of age and urine retention is the humane endpoint (Figure S2). Clearly urinary retention could be restricting movement which would confound behavioural measures. Lastly, it is worth pointing out that many male mouse studies are on mixed, C57BL/6 or C57BL/6J backgrounds, some of which have been performed in different laboratories, which could lead to inconsistencies in outcomes. Overall, we would argue that comparisons of male MPS IIIA mice with WT are not appropriate (or easy to perform) for determination of treatment responses.

Several studies analysed behaviour of male MPS IIIA mice following control intracranial injections. Fraldi *et al.* tested male mice and found that the MPS IIIA mice were hyperactive at 21 weeks of age but not prior to this, but these mice had received intracranial injections and this may have adversely affected the mouse's behaviour [23]. Other studies where intracranial injections have been given to MPS IIIA mice all used male mice and generally observed either no changes or hypoactivity [18], [21], [35]. It is worth considering that intracranial injections could have altered the natural behaviour of the mouse and this may be why hypoactivity is observed.

We found female MPS IIIA mice to be hyperactive, having a significantly increased path length, frequency and duration of rapid exploratory behaviour and reduced duration of immobility at 4 and 6 months of age, (16 and 24 weeks) whilst duration of rapid exploratory behaviour was significantly increased at 8 months (32 weeks). At 4 months of age differences are only significant after 60 minutes, but at 6 months they are all significant after both 10 and 60 minutes. This indicates that the 60 minute test is a more sensitive test to identify hyperactivity than the 10 or 3 minutes tests. No significant differences were observed over the first 3 minutes at any time point, thus we would suggest that studies such as that of Crawley *et al.* 2006 where this short test has been used are not likely to yield significant differences [30].

Our hyperactivity findings in females are supported by Crawley *et al*, where hyperactivity was observed at 22 and 32 weeks of age in a 3 minute test with female MPS IIIA C57BL/6 mice, however no difference was observed at any point prior to 18 weeks or at 25 or 40 weeks of age [30]. They also observed hypoactivity in females at 4 weeks of age but we do not have comparative data to comment on this. The longer testing time utilised in our study may have meant that we were better able to detect behavioural changes in the mice that were not detectable with the 3 minute test.

On the mixed background, female MPS IIIA mice were hyperactive at 3 weeks of age but at subsequent ages no differences were observed at 6, 10, 15, 20, 30 or 40 weeks of age [32]. In the work of Lau *et al.*, using the C57BL/6 backcross, no differences were observed between female WT and MPS IIIA C57BL/6 mice between 3–22 weeks of age [33].

MPS IIIB is a phenotypically indistinguishable disease to MPS IIIA. In the mouse model of MPS IIIB, hyperactivity has been observed in a 10 minute open field test [41] but hypoactivity in an 8 minute open field test performed half in the light half in the dark [42]. A 60 minute open field test did not report differences in path length in male or female mice [43], but a 60 minute open field test that we performed on female mice at the same circadian time as this study observed hyperactivity at 8 months of age [36], which is consistent with our findings here. We also observed significant increases in hyperactivity in male MPS IIIB mice at 8 months of age, although they were less significant differences than those observed in females [14]. The MPS IIIB mouse is a complete knockout and thus may be slightly more severe than the MPS IIIA mouse with ∼3% residual enzyme activity. This may explain why we were able to detect significant genotype differences at 8 months of age in MPS IIIB in all parameters but only some in MPS IIIA. We believe that the 60 minute open field test is still measuring habituation behaviour due to the profiles of activity observed in both MPSIIIA mice in this paper and in MPSIIIB mice [36] over time. We found that differences in behaviour between MPS IIIB and WT mice [36] and MPS IIIA and WT mice (Figure 1B and C) became more pronounced from 3 to 10 to 60 minutes and diverged in the first few minutes from a similar initial response. When we compared MPS IIIB and WT mice over a 24 hour period following 24 hours of habituation [36] the differences were significantly muted and over 14 days there was no significant difference [44]. This suggests that a 60 minute open field test does not reflect home cage behaviour but instead probably measures extended habituation to a novel environment.

A significant decrease in the number of unsupported rears by female MPS IIIA mice was observed at 6 months of age, however, there was no difference in the overall number of rears, or supported rears, at this or any other time point. No consistent trend in the number of rears with female mice is apparent in the literature, Crawley *et al.* reported that female MPS IIIA mice reared less at 15 and 25 weeks of age but there were no significant differences at 9 other time points [30]. No significant differences in rearing were observed in Lau *et al.* who also used backcrossed mice [33], or Hemsley *et al* who used mixed background mice [32]. A more consistent trend is observed in male mice with MPS IIIA rearing less [18], [30], [32], [33], [35], but increased rearing has been observed at 3 weeks [32]. However, at most time points no significant difference is observed. Manual measurement of rears can be subjective and there may be variations in the rearing behaviour, such as amount of time per rear that cannot be measured by simply counting the number of rears. This is why we examined the number of supported and unsupported rears [36], [45]. In the MPS IIIB mouse model, no difference in rearing was observed in the first 10 minutes of an open field test with female mice at 8 months of age [36] and no difference in the first 30 minutes but a significant decrease in the number of rears by 4.5–5 month old male MPS IIIB mice in the second 30 minutes of a 60 minute open field test [43]. Our conclusion is that rearing is too variable an outcome in MPS IIIA and IIIB mice and is therefore not a valuable informative test.

At 4 and 6 months of age the MPS IIIA female mice had a reduced sense of danger and spent a greater proportion of time in the centre of the open field, this measure was significant after 60 minutes but not after 3 or 10 minutes. This is supported by the patients with MPS IIIA which are believed to have a reduced sense of danger [1]. No difference in the time in centre was detected by Lau *et al.* in both male and female mice [33], however this could be because the open field was larger in our study and therefore more sensitive to thigmotaxis [45], [46]. In the MPS IIIB mouse model no differences were observed in the duration spent in the centre of the open field [36] but the open field used in that study was also smaller than the one used in this study.

The elevated plus maze did not demonstrate a decrease in anxiety or sense of danger in the MPS IIIA mice. There were also no differences between repeat tests, and both WT and MPS IIIA mice appeared to habituate equally. Lau *et al.* 2010, observed no significant differences with male mice at 6.5 months (26 weeks) of age with a 5 minute elevated plus maze test, but there was a trend towards reduced anxiety [34]. However in Lau *et al.* 2008 a significant increase in the time the male MPS IIIA mice spent in the open arms at 18 weeks of age, the percentage of the path length on the open arms at 15 and 18 weeks and the percentage of entries into the open arms at 20 weeks was observed [33]. Additionally they observed significant differences between repeats of the elevated plus maze, male MPS IIIA mice had a significantly longer path length in the repeat elevated plus maze test while WT mice remained unchanged [33]. We did not observe decreased anxiety or a change in behaviour between repeat elevated plus maze tests. The width of the arms could be affecting this test as this study used 10 cm wide arms and Lau *et al.*, used 7 cm wide arms. It has been demonstrated that when using 5, 7 or 9 cm wide arms, NMRI mice spent more time in the wider open arms compared to C57BL/10J mice that showed no difference [47]. Ten minutes on the elevated plus maze may also be too short a time to detect the sense of danger differences in the MPS IIIA mice as the only centre measure on open field that is significant after 10 minutes is the number of centre entries in female mice at 6 months of age (p = 0.023). A lack of difference in the elevated plus maze performed in daylight is supported by MPS IIIB mice which showed no difference in the light but were less anxious in the dark [41]. The increased number of centre entries and duration in the open field could perhaps reflect increased but undirected anxiety whereby mice are actually more agitated but have lost the ability to determine what is dangerous. A more pragmatic explanation could be due to the mice not being naively tested as they only received a 30 minute break after the open field test before starting the elevated plus maze test. Prior behavioural tests can affect the result of elevated plus maze tests with C57BL/6J mice that have previously been tested in an open field test [48].

No significant differences were observed in the inverted screen or bar crossing tests which is consistent with the work of Crawley *et al.* who observed no difference in neuromuscular strength with back crossed MPS IIIA mice [30]. However on the mixed background strain, there was a significant decrease in neuromuscular strength with MPS IIIA mice being unable to grip the inverted screen for as long as the WT mice from 20 weeks of age [32]. The work in our study is supported by work with the MPS IIIB mouse model that does not show a decline in motor function at 5 months of age by accelerating rotarod [43], [49] but does by a rocking rotarod test from 9 months onwards [50], [51]. The decline in motor function could relate to the presence of increasing urine retention in the MPS IIIA and B mouse models [14], [26], [29]. Our own tests in the MPS IIIB mouse model found that neuromuscular decline only occurred at 10 months of age (40 weeks) [14], [36]. Given that the MPS IIIA mouse has residual enzyme activity, it is possible that progression of MPS IIIA disease is slightly less rapid than in MPS IIIB which would explain why we did not see a trend to changes in motor function at 8 months in MPS IIIA mice.

Standardisation of tests is a very important aspect of behavioural test design that may have affected comparison of our tests to those of other laboratories. In this study the size of the open field was larger than that described in many studies and the behaviour was observed for much longer (60 minutes compared to 3 minutes). The increased size and time of the test allows better spatial and temporal resolution of the mouse behaviour and so makes the test more sensitive [45]. Video analysis software was used which has been shown to be more sensitive than line crossing determined by an observer or beam breaks [52]. In this study, the mouse was placed in the centre of the open field rather than in the corner and the test was carried out at the same time point of circadian rhythm for all mice as there is a known peak in activity in the similar MPS IIIB mouse model [44]. The mice in this study were also housed differently; females were group housed and male mice were singly housed from 14 weeks of age. The background of the mice can affect the behavioural phenotype, therefore we backcrossed the MPS IIIA mice in this study onto the C57BL/6J background for more than 10 generations. Other groups have used MPS IIIA mice on a mixed background [18], [32], or on a C57BL/6 [23], [30], [33], [34] or C57BL/6J [21] background. Strain specific differences can also significantly affect behavioural outcomes [53].

### Conclusion

We have demonstrated that we can reliably detect differences in the behaviour of female but not male MPS IIIA mice at 4 and 6 months (16 and 24 weeks) of age and these differences match the patient phenotype. Male mice were singly housed due to their aggression which may change comparative behavioural responses. Female MPS IIIA mice are hyperactive, with a longer path length, increased frequency and duration of rapid exploratory behaviour, and spend less time immobile. They also show a trend to this behaviour at 8 months of age with significantly increased duration of rapid exploratory behaviour. Female MPS IIIA mice demonstrate a reduced sense of danger with a greater proportion of the time spent in the centre of the open field with a greater number of centre entries. These two time points and multiple measures are ideal to evaluate novel therapies for MPS IIIA as the effect of a therapy can be sensitively determined using multiple measures. This behavioural phenotype is supported by both the phenotype of the patients and our recent similar observations in the MPS IIIB mouse model [14], [36]. The increased size of the open field, the increased length of the test, the use of video analysis software and performing the test at consistent point in the circadian rhythm have produced a sensitive and robust test to evaluate the effect of therapies on female MPS IIIA mice.

## Supporting Information

Figure S1 **Repeat elevated plus maze behaviour.** At 4, 6 and 8 months of age 7–11 WT male (light grey bars), MPS IIIA male (dark grey bars), WT female (white bars) and MPS IIIA female (black bars) were placed on the elevated plus maze (A) and the behaviour was recorded for 10 minutes. After a 30 minute rest, the test was repeated. The results of the repeat test are presented as the mean of each measure with error bars representing the SEM. p values were calculated by 2 Way ANOVA. The following measures have been presented; the percentage of total entries that were into the open arm, the percentage of the path length in the open arm and the duration spent on the open arm at 4 (A), 6 (B) and 8 (C) months of age.
(TIF)

Figure S2 **Urine retention at 8 months.** At 8 months of age 9 WT male (light grey bars), 8 MPS IIIA male (dark grey bars), 7 WT female (white bars) and 8 MPS IIIA female (black bars) were sacrificed and the urine volume in the bladder measured. The results are presented as the mean with error bars representing the SEM. p values were calculated by 2 Way ANOVA.
(TIF)

Video S1 **Open field behaviour video.** A video running at 4 times the normal speed showing the median female WT and MPS IIIA mouse at 6 months of age. The MPS IIIA mouse is on the left and the WT mouse is on the right.
(WMV)
