# Supplementary figures and images for: Female Mucopolysaccharidosis IIIA Mice Exhibit Hyperactivity and a Reduced Sense of Danger in the Open Field Test
Source: PLoS One. 2011 Oct 18;6(10):e25717. doi: 10.1371/journal.pone.0025717 (PMC3196509; doi:10.1371/journal.pone.0025717)

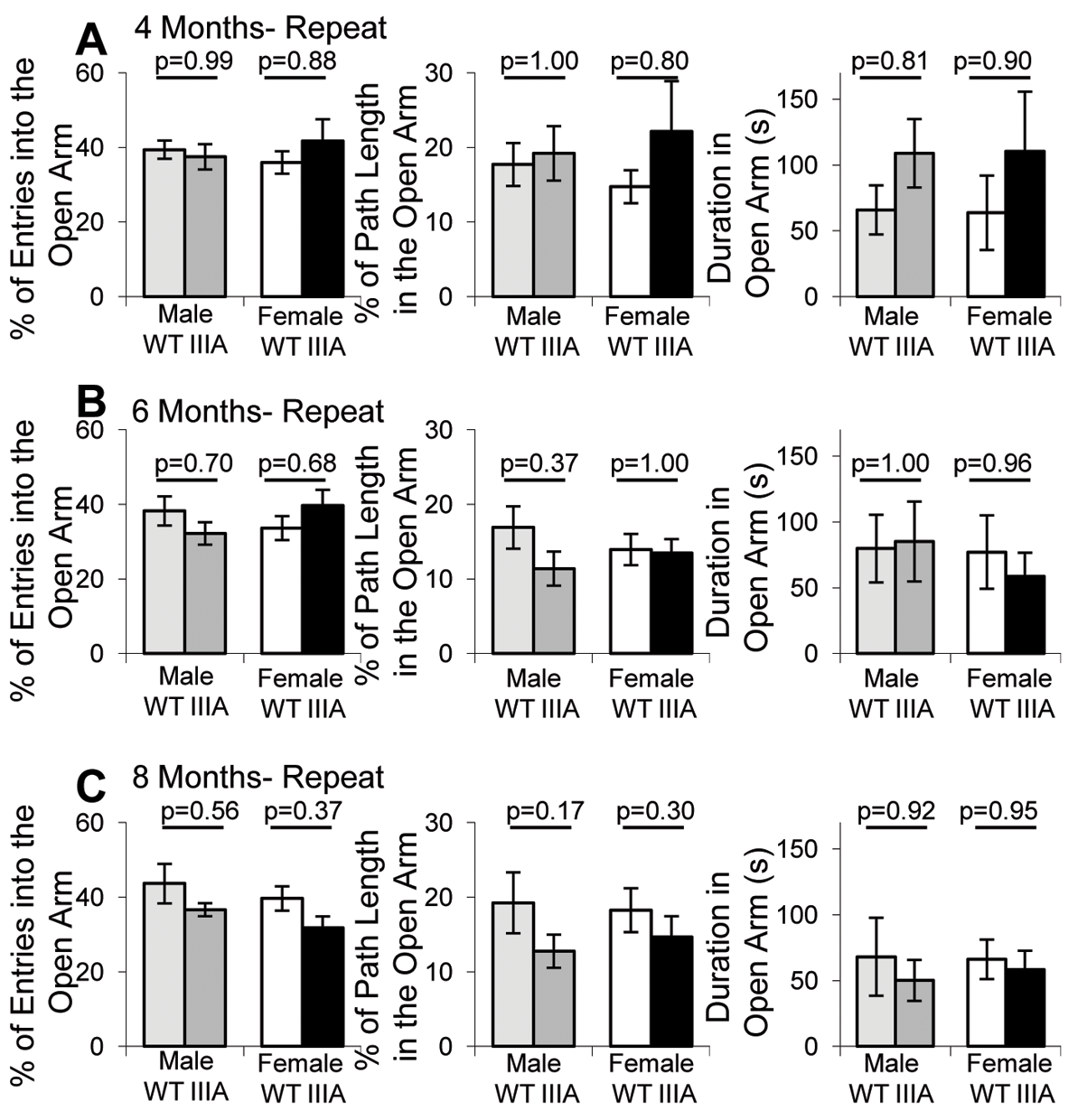

Supplement: Figure S1 — Repeat elevated plus maze behaviour. At 4, 6 and 8 months of age 7–11 WT male (light grey bars), MPS IIIA male (dark grey bars), WT female (white bars) and MPS IIIA female (black bars) were placed on the elevated plus maze (A) and the behaviour was recorded for 10 minutes. After a 30 minute rest, the test was repeated. The results of the repeat test are presented as the mean of each measure with error bars representing the SEM. p values were calculated by 2 Way ANOVA. The following measures have been presented; the percentage of total entries that were into the open arm, the percentage of the path length in the open arm and the duration spent on the open arm at 4 (A), 6 (B) and 8 (C) months of age. (TIF) [file pone.0025717.s001.tif]

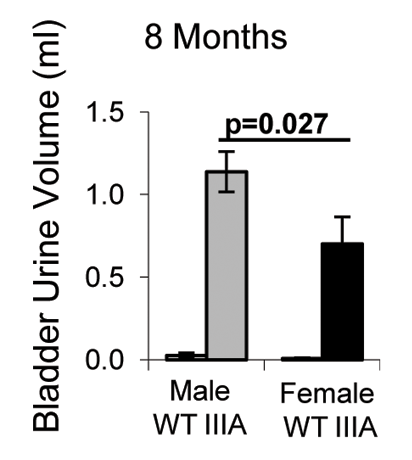

Supplement: Figure S2 — Urine retention at 8 months. At 8 months of age 9 WT male (light grey bars), 8 MPS IIIA male (dark grey bars), 7 WT female (white bars) and 8 MPS IIIA female (black bars) were sacrificed and the urine volume in the bladder measured. The results are presented as the mean with error bars representing the SEM. p values were calculated by 2 Way ANOVA. (TIF) [file pone.0025717.s002.tif]
